# Supplementary material for: Variation in Rice Plastid Genomes in Wide Crossing Reveals Dynamic Nucleo–Cytoplasmic Interaction
Source: Genes (Basel). 2023 Jul 8;14(7):1411. doi: 10.3390/genes14071411 (PMC10379430; doi:10.3390/genes14071411)
Supplement: Supplementary file 1 [file genes-14-01411-s001.zip › Supplementary information 1.pdf]

All primers used in the experiment

Name	Sequence (5' to 3')
CRS1ab-F	TCAGATTGAGCCGAGTTT
CRS1ab-R	TCACGATTGTGCCTGTTA
CRS1cd-F	TCGGGATTCACTAAGACA
CRS1cd-R	TTACGACCTTTACCACAA
CRS2ab-F	CGGGATTCCTCTATTCTA
CRS2ab-R	TCACGATTGTGCCTGTTA
CRS2cd-F	GCATAGGAGGGAAAGTCG
CRS2cd-R	GAATAACCCGGTTGAAGG
CRS3ab-F	AAACCCTGAACCAGGAGC
CRS3ab-R	AAGCGGATTTGTTGAAGC
CRS3cd-F	CAATACATCTCGCGGTAA
CRS3cd-R	TCATCAGCTATGGGTTCA
CRS4ab-F	CGATAACGGGACATGAAG
CRS4ab-R	ATTTGTAATGCGAGGGTC
CRS4cd-F	ATAATGATGGACTGAAAA
CRS4cd-R	CGGACGAATAATCTAATA
CRS5ab-F	AAATGTGCGATATGTTGG
CRS5ab-R	GAGTGGACTCTAGGGATA
CRS5cd-F	GGAGTGGACTCTAGGGATA
CRS5cd-R	AAATGTGCGATATGTTGG
CRS6ab-F	GGCTCGGTTATTCTATCT
CRS6ab-R	CGTATGGCTATGTTCTATTT
CRS6cd-F	AACAGAAATGGATTAACCCTAG
CRS6cd-R	AAGGCGTAGCATTGGAACTG
CRS7ab-F	ATGGGAAATGAAAGTGGC
CRS7ab-R	CCCTGCTTCGCTTAGTGT
CRS7cd-F	AGGTTAGAGCATCGCATTT
CRS7cd-R	TCCTGGCTTCTCCATTTA
CRS8ab-F	GCCTCATCTCGAACAAAT
CRS8ab-R	ATCAGAATCAATCGCTCA
CRS8cd-F	TCTATACTAGGACCTTTACC
CRS8cd-R	CCATATTAGGCTATTTACAC

action-F	AGACCACCTACAACTCTATCA
action-R	TTCCTCCACTAAGCACAA
rpl33-F	AGTCGGCAGGGATTTCTA
rpl33-R	TTTCGGCATGAATCGTAT
psbZ-F	AGTCATTAGTGTACCCCTTG
psbZ-R	GAAAGACCAGTCCAATCC
psbK-F	TTATGCGATTTTCAACCC
psbK-R	TCATCGAAAACTTACAGC
atpF-F	AATGCCACTTCTATTAGTTTGG
atpF-R	GCTCCTTGTACGGCTTGT
ndhD-F	TTTTGGCTTCCTTATTGC
ndhD-R	CGTATGCTCCCATCTTTA
petA-F	CTTGGGTAAAGGAACAGA
petA -R	CAGGCTTATTCGCTAAAT
matK1351-F	ACTCAACCGCAAAGGATC
matK1351-R	CAAATACCAAATACGCTCA
ndhA1070-F	GAGGAAGAATTAGTAGCA
ndhA1070-R	TATAGTGAAACAAGTTGG
rpoB-545-F	TATTGCTAAGTCCTGGTAT
rpoB-545-R	AGTTTGTGGAGTCGGTTT
