# Supplementary material for: Variation in Rice Plastid Genomes in Wide Crossing Reveals Dynamic Nucleo–Cytoplasmic Interaction
Source: Genes (Basel). 2023 Jul 8;14(7):1411. doi: 10.3390/genes14071411 (PMC10379430; doi:10.3390/genes14071411)
Supplement: Supplementary file 1 [file genes-14-01411-s001.zip › Supplementary Tables.pdf]

Table S1. Summary of sequencing plastomes by Illumina

Sample	Clean reads	Clean bases (bp)	Read length (bp)	Sequencing depth (×)
O. glaberrima	777,259	101,593,300	125/150	~755
P10	895,911	134,386,650	150	~999
P88	1,001,710	150,256,500	150	~1116
P90	1,827,040	274,056,000	150	~2036
P91	232,567	34,885,050	150	~259
P92	259,070	38,860,500	150	~289

Table S2. Summary of sequencing plastomes by PicBio

Sample	Clean reads	Clean bases (bp)	Average read length (bp)	Sequencing depth (×)
O. glaberrima	673	8,107,631	12,047	~60
P10	119	1,607,928	13,512	~12
P88	254	3,477,260	13,690	~26
P90	657	8,807,742	13,406	~65
P91	186	2,000,430	10,755	~15
P92	104	1,452,360	13,965	~11

Table S3. All repeats detected in *Oryza glaberrima* plastome

Repeat name	Start site	End site	Length	Distance from the nearest genes
R01	82656	82746	91	⩽2 Kb
R02	55851	55941	91	⩽2 Kb
R03	82882	82941	60	⩽2 Kb
R04	55792	55850	60	⩽2 Kb
R05	44495	44523	29	⩽2 Kb
R06	7833	7861	29	⩽2 Kb
R07	19882	19907	26	>2 Kb
R08	14449	14474	26	⩽2 Kb
R09	46084	46106	23	⩽2 Kb
R10	45575	45597	23	⩽2 Kb

R11	17388	17409	22	≤2 Kb
R12	17352	17373	22	≤2 Kb
R13	44498	44518	21	≤2 Kb
R14	11513	11533	21	≤2 Kb
R15	15109	15129	21	≤2 Kb
R16	45721	45741	21	≤2 Kb

Table S4. Category of multi-copy repeats in *Oryza glaberrima* plastome

Repeat	Length (bp)	Start site	End site
CRS1-1	91	82656	82746
CRS1-2	91	55851	55941
CRS2-1	60	82882	82941
CRS2-2	60	55792	55850
CRS3-1	29	44495	44523
CRS3-2	29	7833	7861
CRS4-1	26	19882	19907
CRS4-2	26	14449	14474
CRS5-1	23	46084	46106
CRS5-2	23	45575	45597
CRS6-1	22	17388	17409
CRS6-2	22	17352	17373
CRS7-1	21	44498	44518
CRS7-2	21	11513	11533
CRS8-1	21	15109	15129
CRS8-2	21	45721	45741

Table S5. RNA editing events detected in plastid of BIL lines

Category	Editing events	Number	Percentage
Nucleotide transition	C-to-U	52	70.3%
	U-to-C	22	29.7%
Edited triplet site	1st	14	18.9%
	2nd	34	45.9%
	3rd	26	35.1%
Amino acid alteration	Synonymous	23	31.1%
	Nonsynonymous	51	68.9%

Table S6. Confirmation of RNA editing using Sanger sequencing

Examined editing sites	*O. glaberrima*			BIL lines			Tested line
	Editing rate by RNA-Seq	Edited clone	Editing rate	Editing rate by RNA-Seq	Edited clone	Editing rate	
matK-1351	30.8%	9/50	18%	80.0%	33/50	66%	P91
ndhA-1070	20.9%	12/50	24%	62.1%	40/50	80%	P90
rpoB-545	32.2%	36/50	72%	57.1%	40/50	80%	P10

Note: In the Sanger sequencing, each editing site were sequenced 50 separate clones.
