# Supplementary figures and images for: Variation in Rice Plastid Genomes in Wide Crossing Reveals Dynamic Nucleo–Cytoplasmic Interaction
Source: Genes (Basel). 2023 Jul 8;14(7):1411. doi: 10.3390/genes14071411 (PMC10379430; doi:10.3390/genes14071411)

***O. glaberrima***

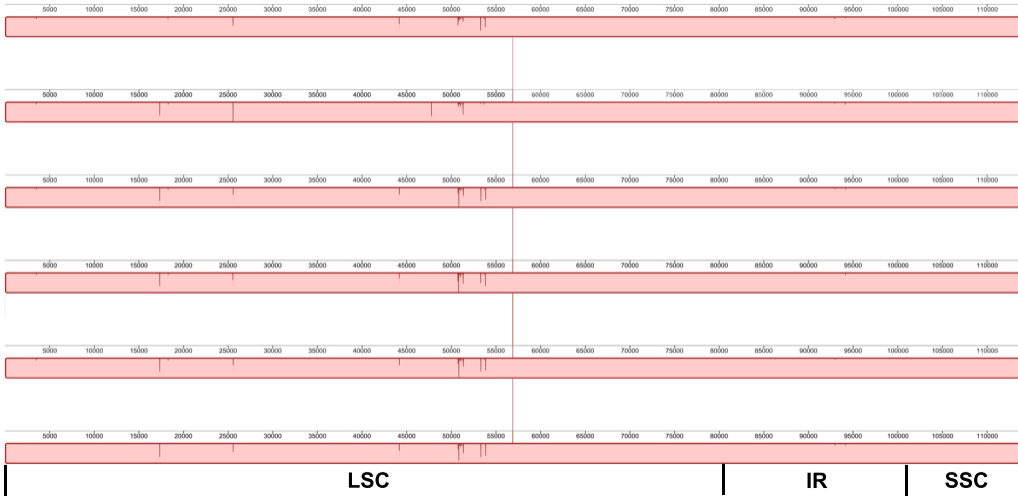

Supplement: Supplementary file 1 [file genes-14-01411-s001.zip › Figure S1.pdf]

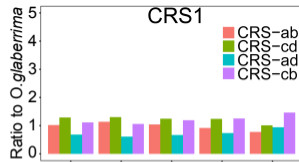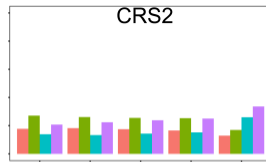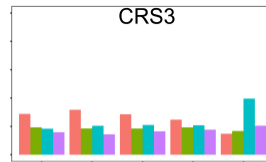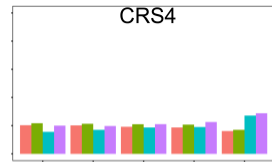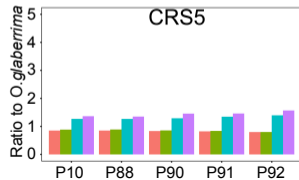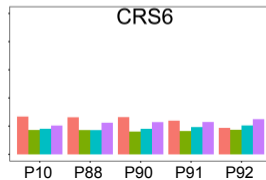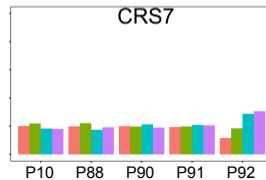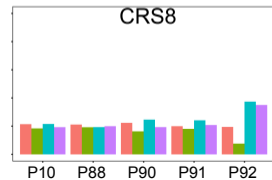

Supplement: Supplementary file 1 [file genes-14-01411-s001.zip › Figure S2.pdf]

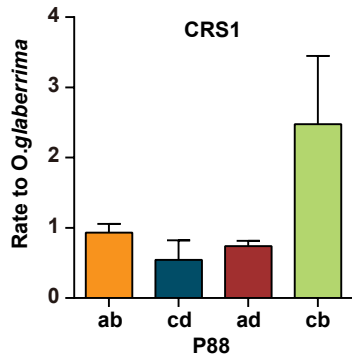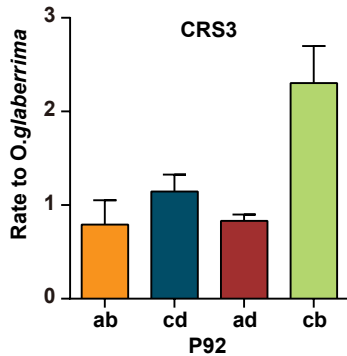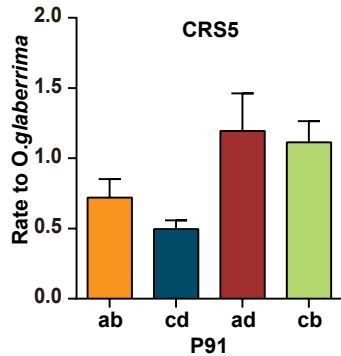

Supplement: Supplementary file 1 [file genes-14-01411-s001.zip › Figure S3.pdf]

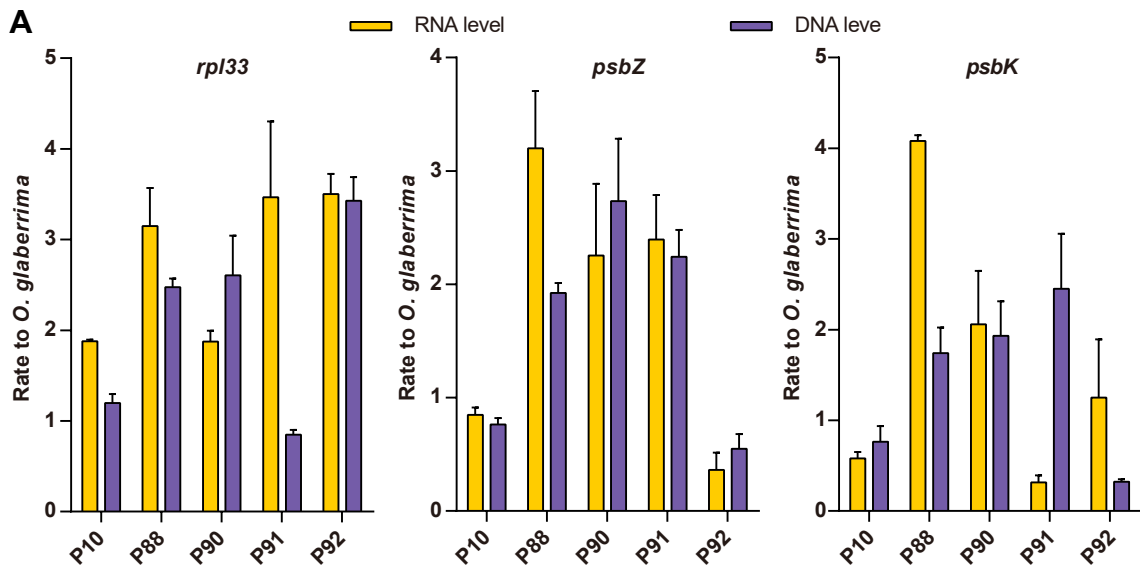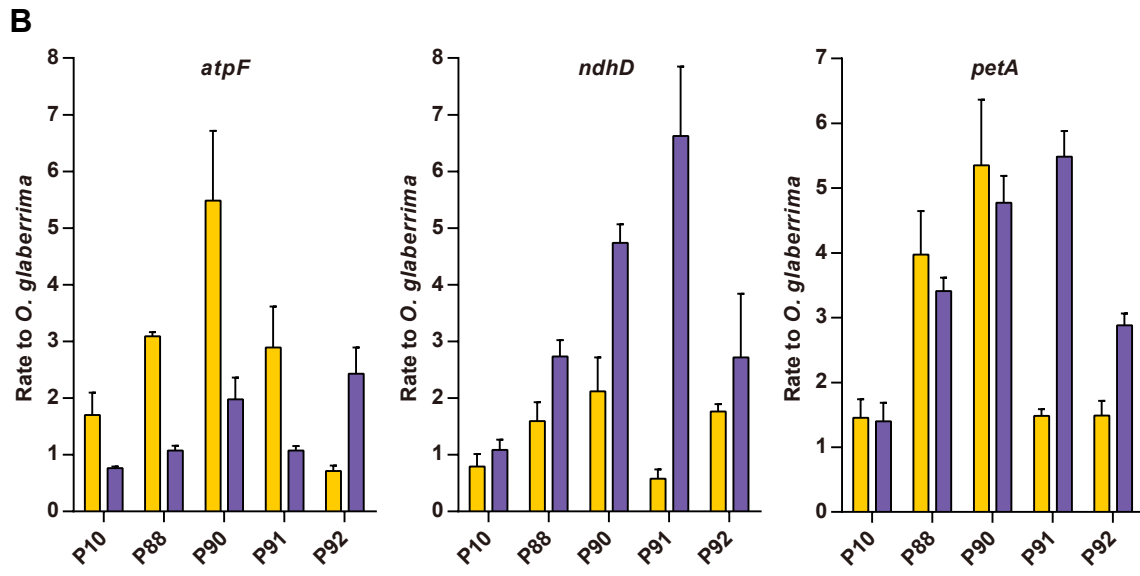

Supplement: Supplementary file 1 [file genes-14-01411-s001.zip › Figure S4.pdf]
